# Stress and anxiety in nursing students between individual and peer simulations

**DOI:** 10.1002/nop2.680

**Published:** 2020-11-18

**Authors:** Natsuki Nakayama, Harumi Ejiri, Naoko Arakawa, Tsuneko Makino

**Affiliations:** ^1^ Department of Integrated Health Sciences Graduate School of Medicine Nagoya University Nagoya Japan; ^2^ Department of Nursing College of Life and Health Sciences Chubu University Kasugai Japan

**Keywords:** anxiety, heart rate variability, peer learning, simulation practice

## Abstract

**Background:**

The use of high‐fidelity simulation practice as an educational tool is becoming increasingly prevalent in nursing education. Despite the learning effects of simulation practice, students have been shown to experience high levels of stress and anxiety during simulation. In recent years, peer learning has been defined as an acquisition of knowledge and skills through active support and support among equal or equal peers and has been shown to be an effective educational intervention for clinical health science students.

**Aim:**

The purpose of this study was to incorporate peer learning into simulation learning and to clarify the differences between stress and anxiety during personal and peer simulations.

**Method:**

Third‐grade undergraduate students in a four‐year course at two nursing universities participated in this study. In this study, the simulated patient was a 53‐year‐old man who had undergone gastrectomy for the treatment of gastric cancer. The scenario was that the patient had completely recovered consciousness in the operating room, and his tracheal tube had been removed one hour before the students examined him. Stress while simulation training was evaluated with heart rate variability. Anxiety was evaluated by the STAI after the simulations were complete.

**Results:**

Personal simulation practice (personal group; *n* = 50) and peer simulation practice (peer group, *n* = 59) was conducted. The personal group included 7 male students, and the peer group included 12 male students; the difference in male proportion was not significant. At the first patient assessment phase, stress of heart rate variability components at the peer group significantly increased relative to that of the personal. In addition, the personal had a significantly higher state anxiety score after simulation than the peer.

**Conclusion:**

This study shows that in the face‐to‐face scene involving vital sign measurements, the presence of peers did not objectively alleviate stress.

## 1 INTRODUCTION

The use of high‐fidelity simulation practice as an educational tool is becoming increasingly prevalent in nursing education. In previous studies, simulation‐based nursing education interventions have been shown to have a strong educational effect, especially in the psychomotor region (Kim et al., 2016; Shin et al., 2015) and to improve nursing student performance (Unsworth et al., 2016).

### 1.1 Background

Student physiological stress (cortisol level) has been found to be increased during simulation practice (McGuire & Lorenz, 2018). In addition, physiological stress responses (heart rate (HR) and cortisol) to practice have been found to be equivalent between the hospital environment and simulation practice (Judd et al., 2016). These previous studies showed that both simulation practices and hospital practices caused high levels of stress. Thus, despite the learning benefits of simulation practice, students experience high levels of stress during simulations. We previously conducted one‐on‐one simulation practice, as one of the traditional methods of simulation practice, to assess and care for patients. However, in one‐on‐one simulation practices, students have reported experiencing stress and anxiety (Nakayama et al., 2018).

Stress is an integral part of nursing student life and education. Students who show high levels of stress have stated that they need to benefit from psychosocial interventions (Rayan, 2019). In addition, stress and anxiety have profound effects on learning through the activation of anxiety hormones that target relevant receptors in working memory (Sanders et al., 2017). It has been observed that in simulation practice, low levels of anxiety lead to optimal performance (Al‐Ghareeb et al., 2019). Studies have shown that stress and anxiety control are important for effective learning in simulation practices.

In a previous study, Guay et al. (2003) showed that peer autonomy and relatedness play key roles in influencing adolescents' use of positive coping behaviours to mitigate stress. Peer learning can be defined as the acquisition of knowledge and skill through active help and support among equal‐status or matched companions, and it has been shown to be an effective educational intervention for health science students in the clinical setting (Secomb, 2008; Topping, 2005). Therefore, we focused on peer learning as a learning method to decrease stress and anxiety. Our research question examined peer simulation practices using peer learning could reduce stress and anxiety more than individual simulation practices. The purpose of this study was to incorporate peer learning into simulation learning and to clarify the differences in stress during simulation practice and anxiety after simulation practice between individual (one‐on‐one) simulation and peer learning simulation.

## 2 MATERIALS AND METHODS

### 2.1 Design

This study is an observational study of students who participated in two types of educational simulations.

### 2.2 Participants

Third‐grade undergraduate students in a four‐year course at two nursing universities participated in this study. We recruited participants by disclosing this study protocol to all students. Only students who indicated their intention to participate were included as participants. All participating students had completed their basic lecture‐based units in basic medicine and basic nursing such as nursing theory and nursing skills before the beginning of this study. None of the students had previously used a high‐fidelity manikin. Each had practiced daily care, such as bathing and shampooing, of one patient over 2 weeks during clinical practice. The students were not accustomed to observing actual perioperative patients. Therefore, the educators prepared simple scenarios that were easy for the students to address. Participants with hypertension, heart disease, diabetes, kidney disease, or reliance on daily medications were excluded from the study. All participants avoided alcohol and caffeine the day before the study and were asked to attempt to sleep well the night before. In addition, participants provided verbal confirmation of the quality of their sleep the day before their study participation. In 2014 and 2015, the individual simulation practice was performed (individual group; *N* = 50). In 2016, 2017 and 2018, the peer learning simulation practice was conducted (peer group, *N* = 59). The individual simulation was performed only for this study, and the peer learning simulation was included in one of the practices. All students were recruited before the two simulations started. In the peer learning simulation, only those willing to participate in this study were included through peer matching.

### 2.3 Simulation scenario

In this study, two types of simulation practices, that is individual and peer, were carried out under the same scenario. The simulation setting was used to help students to learn to observe postoperative patients and to evaluate them using available information. A patient was simulated using a high‐fidelity manikin used for advanced life support training (Laerdal Co., Ltd.). The manikin can emit (and attenuate) a breathing sound and can be set to maintain or change a specific HR, blood pressure and respiratory rate. In this study, the simulated patient was a 53‐year‐old man who had undergone gastrectomy for the treatment of gastric cancer. The scenario was that the patient had completely recovered consciousness in the operating room and his tracheal tube had been removed one hour before the students examined him. When the student examined the patient, he had a central venous catheter, an oxygen mask with a flow rate of 3 L/min, a nasogastric tube, a urethral catheter, a dressing to protect his abdominal wounds and an indwelling abdominal drain. The educator set the physiological parameters of the ECG monitor and manikin as follows: HR 62–70 beats per minute (bpm), blood pressure 120 to 128/66 to 70 mm Hg and respiratory rate 16–20 respirations per minute. In both types of simulation practices, students were given instructions for the simulation scenario before the simulation started. Table 1 shows the progression and differences of the individual and peer simulations phases. The peer simulation consisted of 2–3 groups (4–6 students). In Phase 3, each student performed patient assessment in both types of simulations. In Phase 4 of the peer simulation, student peers exchanged patient information with each other and explained their patient evaluations, which made up for lack of practice.

**Table 1:** Simulation phases and differences between individual and peer simulations

Phases	1	2	3	4
Time, min	5	5	10	5
Contents	Students were given basic information on the patient and setting	Students were given a break	Student examined the manikin through auscultation, inspection and palpation	Student reported on the patient assessment
Individual simulation			Patient assessment by each student	Each student reported his or her patient assessment to educators
Peer simulation			Peer simulation consisted of 2–3 groups (4–6 students) Patient assessment conducted by each student	Students participated in peer work on patient assessment with other students who had conducted the examination

### 2.4 Data collection

Before the simulation practices began, the students received an explanation of the characteristics of the high‐fidelity manikin and were free to touch it. The researcher connected each student to the Holter ECG inspection system (GLLERT Lab Tech Co., Ltd.). Participants were given a questionnaire to evaluate their anxiety (State‐Trait Anxiety Inventory‐Form JYZ (STAI‐JYZ)) after they agreed to participate in the study. They answered the questions without the educator present. Later, the students were given the break to stabilize their autonomic nervous systems. During the break, they sat on a chair in a quiet room with the curtains drawn and were advised to take deep breaths. After the break, the students entered the patient area with the educators and began the first patient assessment phase. After the first patient assessment, the students mainly confirmed the patient's breathing frequency, breathing sounds, cyanosis state and peripheral circulation. In addition, they confirmed that the oxygen tube was not bent and that the dose of oxygen was correct. In the reporting phase, the students who participated in the individual simulation reported the condition of the patient to educators and the students who participated in the peer simulation confirmed their observations among the peer members and shared the information using a large whiteboard. The debriefings of both simulations were conducted in a different room and were based on the following five guidelines by Decker et al. (2013): the debriefings should be: (a) facilitated by an individual who is competent in the debriefing process; (b) conducted in an environment that is conducive to learning and supports confidentiality, trust, open communication, self‐analysis and reflection; (c) facilitated by a person(s) who observed the simulated experience; (d) based on a structured framework for debriefing; and (e) congruent with the students’ objectives and the outcomes of the simulation‐based learning experience (Decker et al., 2013). The educator provided feedback about the patient care phase to the student or students.

### 2.5 Heart rate variability

Heart rate variability (HRV), one of the measurements that can be used for objective stress assessment, is calculated from the change in the R‐R wave interval in successive normal ECG signals measured by the Holter electrocardiographic recording system. HRV is widely accepted as an indicator of autonomic nervous activity (Task Force of the European Society of Cardiology the North American Society of Pacing Electrophysiology, 1996). Previous studies have measured stress using HRV (Dishman et al., 2000; Hjortskov et al., 2004). Frequency analysis of the HR (MemCalc GMS) can be used to extract the high‐frequency component (HF: 0.15–0.4 Hz) and the low‐frequency component (LF: 0.04–0.15 Hz), which can be used as indicators of parasympathetic and sympathetic activity (LF/HF) (Punita et al., 2016; Sin et al., 2016). An increase in parasympathetic nervous activity is associated with stress relief and a decrease is related to increased stress (Kim et al., 2018). Thus, educators can measure HRV to assess stress situations during student simulations. In this study, among the individual and peer simulation practices, we extracted HF and LF/HF components at two phases of simulation: (a) a break; and (b) the first patient assessment. We calculated data for each phase by averaging all values of the phase.

### 2.6 State‐Trait Anxiety Inventory‐Form JYZ (STAI)

In this study, participants completed self‐assessments using the STAI‐JYZ to measure anxiety. The STAI‐JYZ was created based on Spielberger's STAI (Gaudry et al., 1975). The reliability and validity of the STAI‐JYZ have been confirmed, and the scale is widely used (Shimizu & Imae, 1981). The STAI‐JYZ is able to measure state‐trait anxiety. State anxiety assesses "How do you feel now?" and indicates a transient response to events that cause anxiety. Trait anxiety assesses "How do you usually feel?" Trait anxiety is a stable trait relative to state anxiety that recognizes various threat situations in the same way and responds in the same way. In this study, state anxiety and trait anxiety were measured before the start of simulation practice (before) and state anxiety was measured again after the simulation practice (after).

### 2.7 Analytical methods

Data from the two groups were compared using the chi‐square test and the Mann–Whitney *U* test using SPSS v.25 software (IBM Corp.). The HR, HF and LF/HF components in the two phases (the break and patient assessment phases) were compared between the individual and peer simulation practices. The statistical significance level was set at *p* < .05.

### 2.8 Ethical considerations

The educator obtained informed consent from all participants and explained the ethical considerations of participating in the study as follows: participation was voluntary, and the students could withdraw from the study at any time without affecting their grades. This study was conducted with the approval of the research ethics committee of the university that conducted the study.

## 3 RESULTS

**Figure 1:**
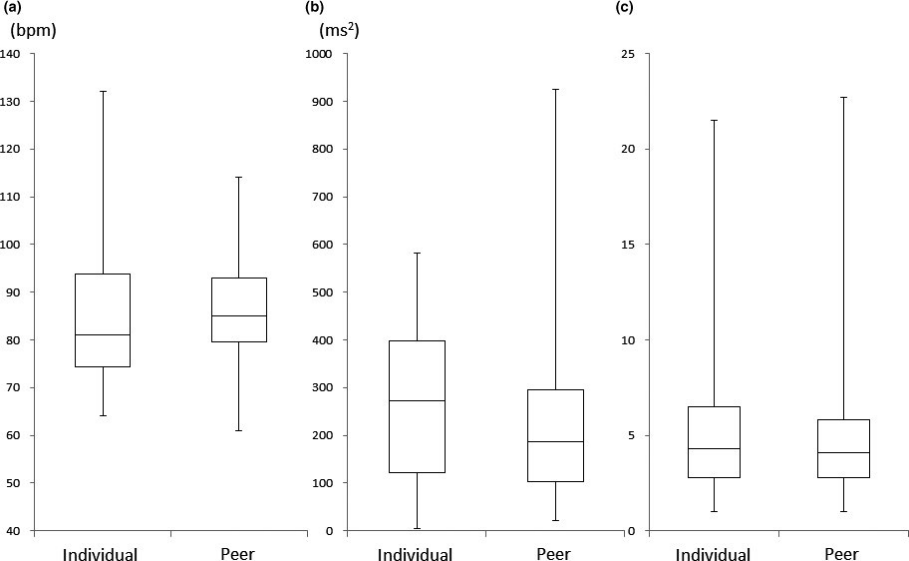
Comparisons between the Individual Group (*n* = 50) and Peer Group (*n* = 59) during the Break. (a) Heart rate, (b) High‐frequency component, (c) Low frequency/High frequency

**Figure 2:**
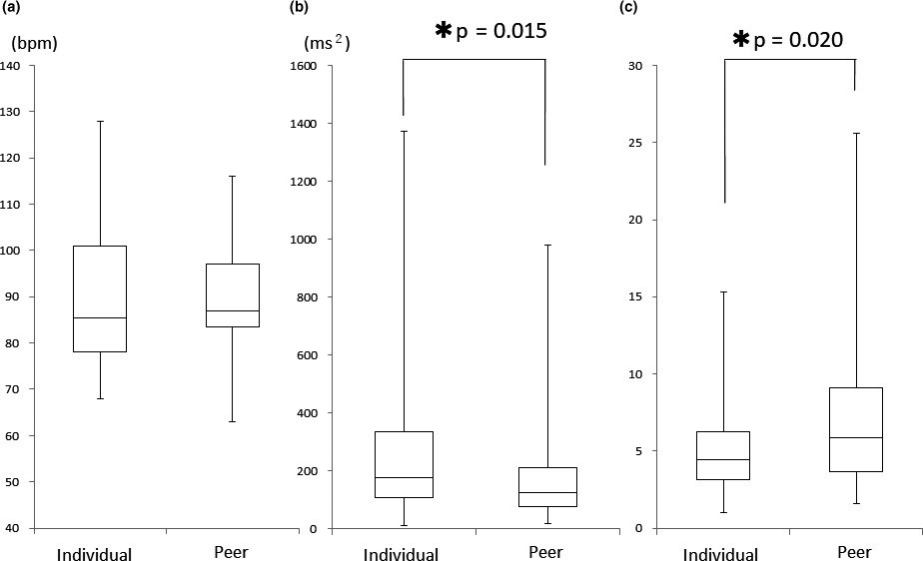
Comparisons between the Individual Group (*n* = 50) and Peer Group (*n* = 59) during the First Patient Assessment. (a) Heart rate, (b) High‐frequency component, (c) Low frequency/High frequency *p < .05

**Figure 3:**
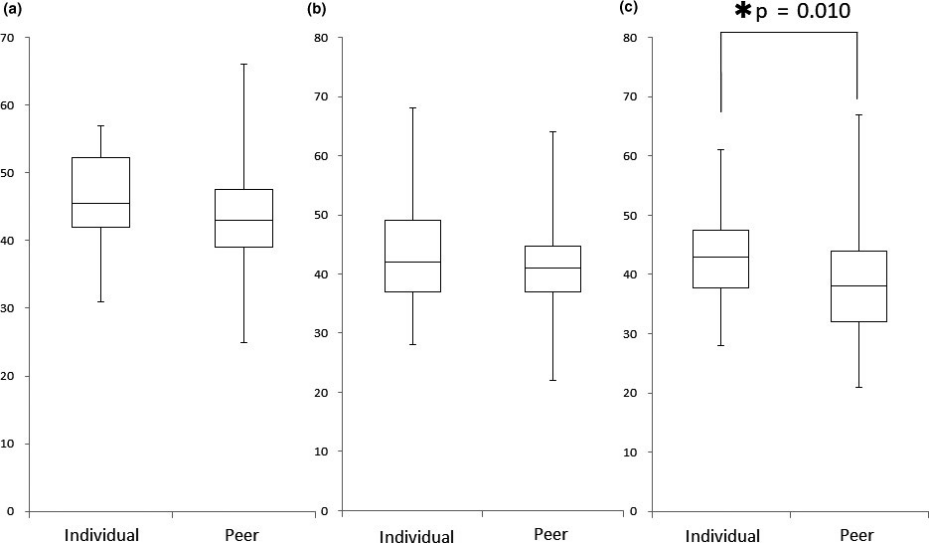
Comparison of STAI Scores between the Individual Group (*N* = 50) and Peer Group (*N* = 59). (a) STAI trait anxiety items, (b) STAI state anxiety items before simulation practice, (c) STAI state anxiety items after simulation practice *p < .05

In this study, 50/212 (23.5%) students participated in the individual simulation and 59/318 (18.5%) students participated in the peer simulation. The individual group included 7 male and 43 female students and the peer group included 12 male and 47 female students; the difference in the proportion of males between the two groups was not significant (*p* = .453). The median age of participants in the two groups was 21 years (range 20–22 years), and no significant differences were observed between the two groups in terms of background (Table 2). In addition, the participants in each peer group were in the same grade. We compared the HR, HF and LF/HF components at the break and the first patient assessment. No significant difference was observed in the HR, HF or LF/HF component between the two groups during the break phase (*p* = .133, 0.104, 0.942) (Figure 1). During the first patient assessment phase, no significant difference was found in HR between the two groups (*p* = .263) (Figure 1), but the HF component of participants in the peer simulation was significantly decreased relative to that of participants in the individual simulation (*p* = .015) (Figure 3). In addition, the LF/HF component was significantly increased under peer simulation relative to individual simulation (*p* = .020) (Figure 2).

**Table 2:** Background of the individual group and peer group

	Individual group (*n* = 50)	Peer group (*n* = 59)	*p* value
Female^a^	43	47	.453
Man^a^	7	12	
Age, median (range)^b^	21 (20–22)	21 (20–22)	.853

^a^ Chi‐square test.

^b^ Mann–Whitney *U* test.

The scores of state and trait anxiety before practice as obtained by the STAI did not differ between the individual and peer simulations (*p* = .352, 0.077; Figure 3). However, after simulation, the individual group had a significantly higher state anxiety score than the peer group (*p* = .010; Figure 3).

## 4 DISCUSSION

In this study, during the first patient assessment phase, no significant difference in HR was found between the two types of simulation, but the HF index was significantly decreased and the LF/HF index was significantly increased in the peer simulation group relative to the individual simulation group. Thus, the peer simulation students were exposed to greater stress at the patient assessment phase than were the individual simulation students. In previous studies, nursing students were found to experience greater stress and pressure during simulation practice than during passive learning (Boostel et al., 2018; Feingold et al., 2004; Lasater, 2007). In the patient assessment phase, students practice face‐to‐face approaches such as feeling a pulse, observing chest movements, listening to respiration sounds and measuring blood pressure (Eyikara & Baykara, 2018). "Vital sign" measurement through a face‐to‐face approach is an important topic in nursing education that involves the acquisition of cognitive and psychomotor skills (Gordon et al., 2013). The results of this study suggest that the peer simulation students might have been more concerned about their lack of knowledge and skills than were individual simulation students due to the sharing of a patient at the same time. This study showed that the presence of peers did not objectively reduce stress in the patient assessment phase, which included vital sign measurement.

Preparing for stressful situations can be achieved not only by the practice of skills and knowledge but also by positive cognitive preparation and encouragement from team members (vonRosenberg, 2019). Positive perceptions and encouragement from team members focus on the issues rather than the threats of the situation and can encourage health care providers to address issues positively, increase their confidence and improve their performance (vonRosenberg, 2019). Therefore, the stress of peer simulations identified in this study may help to stimulate performance. Facilitators may be able to facilitate communication between peers who share the same patient at the same time, establish positive cognition, promote performance and operate peer simulation more effectively.

In this study, there was no difference between the two groups in state or trait anxiety, as measured using the STAI, before undergoing simulation. However, in postsimulation state anxiety, the individual simulation students scored significantly higher than the peer simulation students. This result indicates that the anxiety level in the individual simulation group was higher than that in the peer simulation group postsimulation. Previous studies have shown that anxiety adversely affects learning (Jiang et al., 2018) and negatively affects nursing students' experience in the field of clinical nursing (Pai, 2016). These previous studies suggested that high values of state anxiety might not lead to efficient learning effects of simulation. In addition, active support from fellow students in peer learning can improve students' ability to deal with the challenges they face (Christiansen & Bell, 2010). Nursing students reported general satisfaction with peer learning, noting that it allowed them to engage in more detailed learning than traditional learning methods (Ravanipour et al., 2015). In this study, we found that peer simulations can reduce student anxiety relative to that experienced under individual simulation, potentially enabling students to learn more effectively.

This study had the following strengths and limitations. This study showed that nursing students increased stress and reduced anxiety from peer simulation practices. Therefore, even in hospital nursing education practice, nursing students may be able to enhance their learning effectiveness through active support and support among peers in the same nursing student positions. In addition, in the field of nursing practice, peer nursing may help nurses provide care without anxiety. The present study does not reveal any differences in performance between the individual and peer simulations. Future research should focus on the differences in learning effects between different methods.

## 5 CONCLUSIONS

This study compared student stress during individual and group simulation practices. During the patient assessment phase, the stress level of the peer‐learning simulation group was significantly increased relative to that of the individual simulation group. This study showed that in face‐to‐face interactions involving vital sign measurements, the presence of other students does not objectively reduce stress. In addition, the anxiety level of the peer‐learning simulation group was significantly decreased relative to that of the individual simulation group. The findings indicate that the type of simulation method affects the stress situation experienced by students and student anxiety.

## CONFLICT OF INTEREST

The authors declare no conflict of interest associated with this manuscript.

## Data Availability

The data that support the findings of this study are available from the corresponding author, N.N, upon reasonable request.
